# Breast clinicians: their contribution to the breast screening programme in England and implications of the current age structure

**DOI:** 10.1186/bcr3314

**Published:** 2012-11-09

**Authors:** C Swinson

**Affiliations:** 1 Beds & Herts Breast Screening Service, Luton, UK

## Introduction

Since the role of breast clinician was established in 1987 to support introduction of the NHSBSP, the role has developed and breast clinicians are now important members of the MDT, skilled in the full range of screening work. With a shortage of radiologists this input must continue, but recruitment may be hindered by a restrictive contractual structure. This study set out to establish the contribution of breast clinicians to the screening programme, and their age distribution.

## Methods

In July 2012 an email survey of members of the Association of Breast Clinicians was conducted to determine staffing, film-reading workload and age distribution data in English screening units. Responses were received from 16 units, around 90% of units with breast clinician film-readers.

## Results

The 16 units were mainly in the south and included some of the largest screening units. Overall, there were 30 breast clinician film-readers, 56 consultant radiologists and 46 radiographer film-readers. Numbers of clinicians in units varied from one to five, representing 9 to 50% of all film-readers. Breast clinicians read from 9 to 66% of films, and in 12 units read proportionately more films than expected from their number. Twenty-two (73%) of the clinicians were aged over 50 and 14 (47%) over 55.

## Conclusion

Breast clinicians make a substantial contribution to screening units in England, particularly in the south. Retirements over the next 5 to 10 years will add considerably to manpower pressures on the NHSBSP. Recruitment will be dependent upon an attractive career structure and robust training.

